# Chronic Wound Healing by Amniotic Membrane: TGF-β and EGF Signaling Modulation in Re-epithelialization

**DOI:** 10.3389/fbioe.2021.689328

**Published:** 2021-07-06

**Authors:** Catalina Ruiz-Cañada, Ángel Bernabé-García, Sergio Liarte, Mónica Rodríguez-Valiente, Francisco José Nicolás

**Affiliations:** ^1^Laboratorio de Regeneración, Oncología Molecular y TGF-β, IMIB-Arrixaca, Murcia, Spain; ^2^Unidad de Heridas Crónicas y Úlcera de Pie Diabético, Hospital Clínico Universitario Virgen de la Arrixaca, Murcia, Spain

**Keywords:** amniotic membrane, wound healing, cell models, TGF-β signaling, EGF signaling, re-epithelialization

## Abstract

The application of amniotic membrane (AM) on chronic wounds has proven very effective at resetting wound healing, particularly in re-epithelialization. Historically, several aspects of AM effect on wound healing have been evaluated using cell models. In keratinocytes, the presence of AM induces the activation of mitogen-activated protein (MAP) kinase and c-Jun N-terminal kinase (JNK) pathways, together with the high expression of c-Jun, an important transcription factor for the progression of the re-epithelialization tongue. In general, the levels of transforming growth factor (TGF)-β present in a wound are critical for the process of wound healing; they are elevated during the inflammation phase and remain high in some chronic wounds. Interestingly, the presence of AM, through epidermal growth factor (EGF) signaling, produces a fine-tuning of the TGF-β signaling pathway that re-conducts the stalled process of wound healing. However, the complete suppression of TGF-β signaling has proven negative for the AM stimulation of migration, suggesting that a minimal amount of TGF-β signaling is required for proper wound healing. Regarding migration machinery, AM contributes to the dynamics of focal adhesions, producing a high turnover and thus speeding up remodeling. This is clear because proteins, such as Paxillin, are activated upon treatment with AM. On top of this, AM also produces changes in the expression of Paxillin. Although we have made great progress in understanding the effects of AM on chronic wound healing, a long way is still ahead of us to fully comprehend its effects.

## Introduction

**FIGURE 1:**
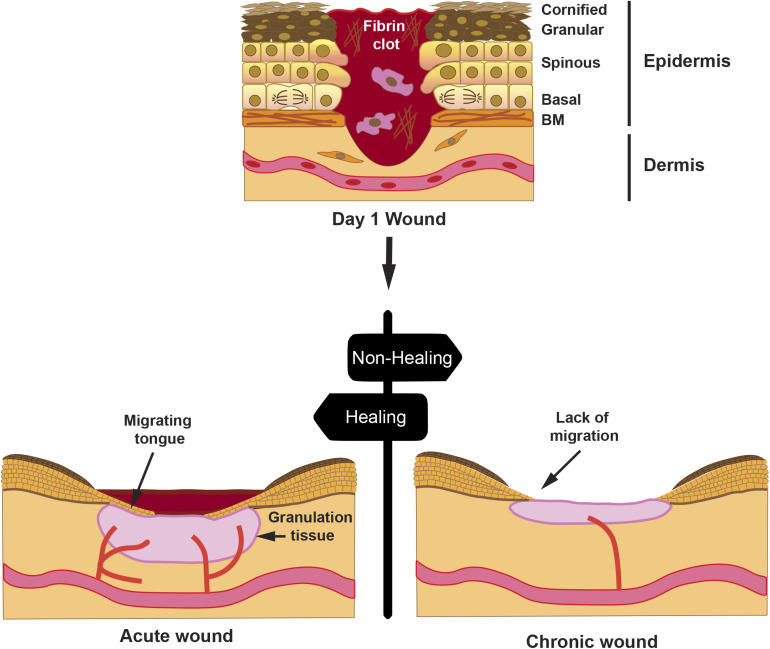
Progression of a wound. A full-thickness wound is depicted at day 1 post-injury; the skin epithelium with its cell layers indicates that the keratinocytes from the spinous layer are activated for migration, whereas keratinocytes from the basal layer proliferate to sustain cell numbers during migration. Hemostasis occurring on day 0 creates a fibrin clot with immune cells, such as macrophages. Later, an acute wound will proceed with healing with reduction of the fibrin clot, formation of granulation tissue, and re-epithelialization by creating a migrating tongue. However, in a chronic wound, granulation tissue is diminished, re-epithelialization is halted, and wound remains open after an extended time. Drawn cells in the dermis represent fibroblasts.

A proper wound healing process in an orderly and timely manner is critical for skin restoration after injury. This process involves four stages, whose progression overlaps, namely, hemostasis, inflammation, proliferation, and remodeling (Singer and Clark, 1999; Insausti et al., 2016; Castellanos et al., 2017). During hemostasis, the blood clot controls the blood flow and establishes a primary matrix into which cells migrate. During the inflammation stage, monocytes and lymphocytes are induced to extravasate toward the wound bed, where they secrete cytokines and growth factors that activate fibroblasts. In the proliferative phase, fibroblasts embedded in the blood clot proliferate and secrete a provisional extracellular matrix (ECM), which contributes to the formation of granulation tissueand new blood vessels sprout to maintain the viability of the new tissue. Concomitantly, keratinocytes lose their contact with the basal lamina and are prompted to migrate toward the wound gap (Figure 1). A re-epithelialization tongue makes its way between the granulation tissue and the wound scab. Finally, during the remodeling phase, wound contracts forming a scar involving ECM remodeling and apoptosis of fibroblasts and macrophages, once their function has been completed.

Various factors, such as, age, diabetes, or large deep wounds, may lead to wound chronification with negative consequences, thus resulting in re-epithelialization failure (Figure 1). Most chronic wounds get stuck in the inflammatory or the proliferative phase (Enoch and Price, 2004). In such situations, it is critical to aid wounds into a proper healing resolution. The use of amniotic membrane (AM) as a dressing has proven successful in resetting chronic wound conditions into a favorable resolution. In addition to the antimicrobial (Valiente et al., 2018) and immunomodulatory effect, AM secretes a set of growth factors, including transforming growth factor (TGF)-β and epidermal growth factor (EGF) (Koizumi et al., 2000; Parolini et al., 2008), which may have a direct function in stimulating the migratory capacity of keratinocytes (Hashimoto, 2000). In keratinocyte *in vitro* wound scratch models, it has been shown that, indeed, AM has a stimulatory effect on migration (Insausti et al., 2010; Alcaraz et al., 2015; Ruiz-Canada et al., 2017). In this review, we will summarize a current view of the AM capability to stimulate re-epithelialization in chronic wounds. We will also discuss identified signaling pathways in epithelial cells, involved at the cellular level, which prompt this AM-stimulated re-epithelialization.

## AM in Chronic Wound Healing

Chronic wounds fail to proceed with wound closure. This occurs for a variety of reasons, including impaired vascularization/oxygenation, deficient cytokine levels, fibrotic and desiccated tissues, etc. In most cases, these factors lead to a long-lasting inflammatory process, an altered proliferative phase, and cell senescence (Castellanos et al., 2017). The benefits of AM in human therapy are well established, from its use as a complete AM dressing to the use of its cell constituents or AM extracts used with some other matrix vehicle (Parolini and Caruso, 2011; Murphy et al., 2017). AM displays anti-inflammatory and anti-bacterial properties, and it also possesses low immunogenicity (Parolini et al., 2009). Several clinical studies have used AM for the treatment of skin wounds, burn injuries, chronic leg ulcers, diabetic foot ulcers, prevention of tissue adhesion in surgical procedures, and ocular surface reconstruction (Parolini et al., 2009; Insausti et al., 2010; Valiente et al., 2018). In all cases, AM has been used in the absence of immunosuppressive treatment without induction of acute immune rejection. AM-secreted factors stimulate fibroblasts and keratinocytes during the proliferative phase. In this regard, it has been shown that chronic wound fluid is less mitogenic, providing a senescent phenotype (Bucalo et al., 1993; Harris et al., 1995). In the case of keratinocytes, AM stimulates their migration leading to the re-epithelialization of the wound (Lee and Tseng, 1997; Insausti et al., 2010). Coincidently, keratinocytes’ migratory capacity, rather than their proliferation, is thought to be reduced in chronic versus acute wounds (Andriessen et al., 1995; Enoch and Price, 2004). Nevertheless, the proliferation of keratinocytes is necessary for proper epithelialization (Yang et al., 2001; Liarte et al., 2020b). Chronic wounds also present with poor microvasculature formation at the wound bed, which leads to hypoxia and low nutrient supply, thus contributing to faulty healing. It has been shown that AM has angiogenic properties, such as the promotion of microvessel formation and recruitment of hematopoietic progenitor cells (Duan-Arnold et al., 2015; Maan et al., 2015). Finally, during the remodeling phase, it has also been shown that AM improves wound contraction and scar formation (Loeffelbein et al., 2012).

## AM Mechanisms of Action on Keratinocytes: *in vitro* Models

Both proliferating and migrating keratinocytes are detected during effective re-epithelialization after wound injury (Garlick and Taichman, 1994). Typically, these two properties are stimulated by the local wound milieu, which shows a particular composition of the ECM and the presence of growth factors and cytokines produced by cells of the granulation tissue and the fibrin clot (Singer and Clark, 1999). In chronic wounds, the milieu fails to provide the appropriate cocktail for wound closure (Barrientos et al., 2008); in this scenario, AM is able to rescue wound closure by providing the appropriate cytokines and growth factors as well as serving as a dressing (Parolini et al., 2009; Insausti et al., 2010). In order to decipher the mechanism by which AM executes this rescue on keratinocytes, we and other researchers have used the human spontaneously immortalized keratinocyte (HaCaT) cell line. The HaCaT cell line is closely approximated to normal keratinocytes and it is capable of forming an orderly and differentiated epidermal tissue when transplanted onto nude mice (Boukamp et al., 1988).

### Role of AM in Migration

Keratinocyte motility is driven by rearrangements of the actin cytoskeleton to produce lamellipodia and filopodia, both of which adhere to the ECM with the help of integrins and drag the cell forward (Mayor and Etienne-Manneville, 2016). During migration, keratinocyte gene expression profile changes to generate a different cell surface set of integrins and increases certain types of secreted matrix metalloproteinases (MMPs) to degrade matrix components (Coulombe, 1997). In addition, urokinase [also known as urokinase plasminogen activator (uPA)] and tissue plasminogen activator (tPA) are expressed to degrade the fibrin eschar (Grondahl-Hansen et al., 1988; Coulombe, 1997). Activator protein-1 (AP-1) transcription factors, of which c-Jun is part, are involved in the transcription of genes, such as integrins and MMPs, among others (Yates and Rayner, 2002).

Amniotic membrane enhances cell migration on *in vitro* HaCaT wound healing scratch assays (Alcaraz et al., 2015; Ruiz-Canada et al., 2017). A landmark of AM action on chronic wounds is the high c-Jun induction at the migratory tongue (Insausti et al., 2010; Alcaraz et al., 2015); its expression at this location is necessary for re-epithelialization of wounds (Li et al., 2003). Strikingly, c-Jun induction is replicated in the migration assay of HaCaT cells stimulated by AM (Alcaraz et al., 2015). Moreover, AM enhances the migratory capacity of Mv1Lu cells, a non-malignant mink lung epithelial cell migration model (Demetriou et al., 1995), by producing the overexpression of c-Jun at the wound healing scratch assay leading edge (Alcaraz et al., 2015; Ruiz-Canada et al., 2017).

**FIGURE 2:**
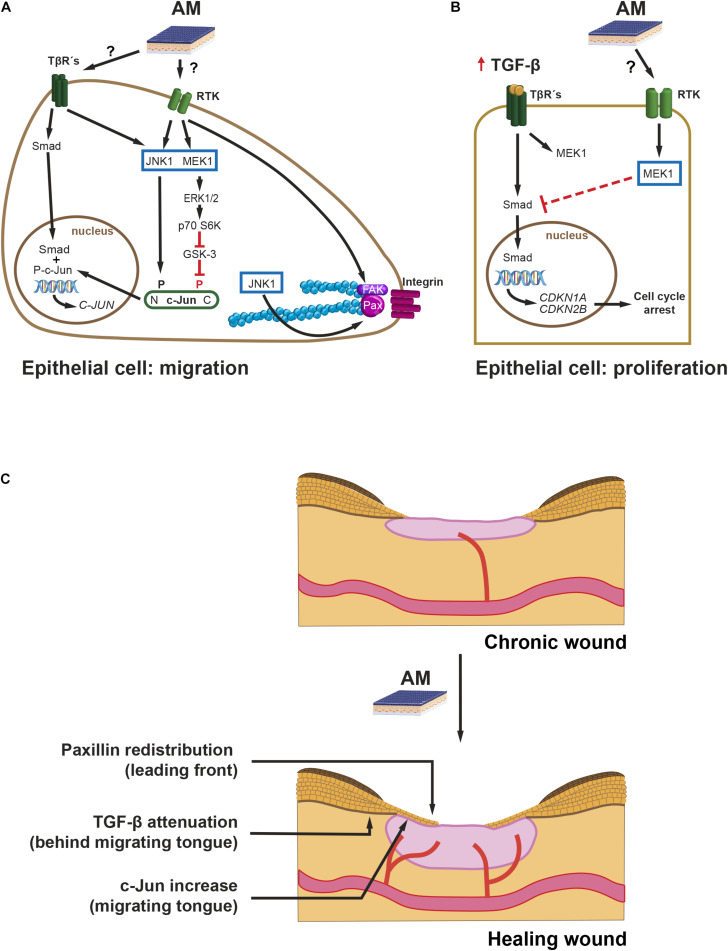
Chronic wounds. Intracellular pathways leading to migration and cell proliferation during AM-stimulated re-epithelialization. Proposed mechanisms of action: **(A)** AM, possibly by secreting EGF and low levels of TGF-β, stimulates JNK1 and MEK1-ERK1/2, which leads to active c-Jun protein. N-terminus-phosphorylated, P, c-Jun, together with nuclear Smad, enhances c-Jun expression. Additionally, AM activation of JNK1 increases remodeling of FS by phosphorylation of Paxillin (Pax). Finally, the stimulation of FAK by AM can also promote cell migration. P70 S6 kinase (p70 S6K). **(B)** An excess of TGF-β induces the expression of cell cycle inhibitor genes: *CDKN1A* and *CDKN2B*. AM induces an attenuation of the Smad phosphorylation mediated by MEK, which prevents proper expression of *CDKN1A* and *CDKN2B* and resumes cell cycle. **(C)** AM proposed mechanism of action on chronic wound re-epithelialization. The effect of AM on halted chronic wound can be summarized by the re-initiation of the migrating tongue that leads to re-epithelialization by the proposed mechanisms: (i) FS remodeling by Paxillin remodeling at the leading front, (ii) overexpression of c-Jun at the migrating tongue, and (iii) attenuation of TGF-β signaling at the rear of the migrating tongue.

From a time perspective, and paying attention to protein expression, c-Jun is c-Jun N-terminal kinase (JNK)-dependent N-terminal phosphorylated in response to AM in the short term (up to 6 h) and remains phosphorylated up to 24 h later at the Mv1Lu cells migration front, with a concomitant rise of c-Jun protein levels throughout (Alcaraz et al., 2015; Ruiz-Canada et al., 2017). Similarly, in HaCaT cells, AM also induces enhanced levels of c-Jun protein in wound healing scratch assays in the long term (Alcaraz et al., 2015). To gain further knowledge of the signaling mechanisms leading to AM activation of c-Jun, we have to consider that the EGF family is involved in the cell proliferation and migration of keratinocytes (Hashimoto, 2000). On the other hand, TGF-β plays a key role in keratinocyte epithelial to mesenchymal transition (EMT) (Liarte et al., 2020a). However, EMT does not reach completion unless HaCaT cells have an enhanced Ras activity, for instance by means of co-stimulation with high doses of EGF (Davies et al., 2005). When the leading edge of a wound healing scratch assay is observed at 24 h (Mv1Lu), c-Jun increases, and its JNK-dependent phosphorylation enhances (Alcaraz et al., 2015; Ruiz-Canada et al., 2017). It is known that EGF receptor (EGFR) signaling can synergize with integrin signaling (Miyamoto et al., 1996; Geiger and Yamada, 2011; Figure 2A). Both cell surface receptors use growth factor receptor-bound protein (Grb) 2/Son of Sevenless (SOS) to activate Ras, which in turn activates JNK kinases and, therefore, phosphorylates N-terminal c-Jun (Figure 2A). The activation of JNK can also be produced by the TGF-β non-Smad signaling branch (Moustakas, 2005). Altogether, AM activation of JNK may account for the short term (6 h) accumulation of c-Jun, because the phosphorylation of c-Jun stabilizes the protein (Musti et al., 1997). Furthermore, in the long term, transcription by phosphorylated c-Jun may sustain the high levels of c-Jun protein. In addition, Smad-dependent transcription activity may contribute to it in the long term, since it synergizes with AP-1 enhancing c-Jun transcription (Wong et al., 1999). Coherently, TGF-β/Smad pathway inhibition by SB431542 inhibitor (Inman et al., 2002) is detrimental for AM-induced migration, suggesting that this signaling contributes to the effect of AM on migration (Ruiz-Canada et al., 2017). Nevertheless, it would be interesting to analyze the possible effect of SB431542 on the AM itself, which has not been fully elucidated (Alcaraz et al., 2013). Moreover, AM migration stimulation is enhanced by the presence of TGF-β (Ruiz-Canada et al., 2017). However, when TGF-β receptor I (TβRI) is not present, the additive effect of TGF-β on AM migration is not observed, which endorses the idea that there is a moderate contribution of Smads to AM migration. This is further supported by the fact that overexpression of Smad2 increases the migration induced by AM (Ruiz-Canada et al., 2017). From another perspective, it is known that extracellular signal-regulated kinase (ERK) activation leads to the removal of phosphorylation at c-Jun carboxy-terminus by glycogen synthase kinase (GSK)-3 kinase (Figure 2A), a necessary event so that c-Jun can bind DNA and be transcriptionally active (Meng and Xia, 2011). It is then plausible that sustained ERK activation by AM may be the mechanism whereby meaningful levels of transcriptionally active c-Jun can be achieved (Figure 2A). In summary, a complex signaling network of events is triggered by AM to activate c-Jun, a master regulator of migration (Li et al., 2003) (Figure 2C).

Regarding the immediate migration machinery, at the cell sheet migrating front, AM induces the remodeling of focal structures (FS) beneath cell protrusions (Bernabe-Garcia et al., 2017). Concomitantly, AM increases protein levels of Paxillin, which is an important scaffold protein that recruits a variety of signaling molecules to FS, namely, focal complexes and focal adhesions. Among these signaling proteins, we can highlight focal adhesion kinase (FAK). FAK is activated by integrin-mediated cell adhesion or growth factors, and it, in turn, activates downstream effectors to regulate cell motility (Sieg et al., 2000; Deakin and Turner, 2008). Paxillin phosphorylation by JNK at Ser 178 (Huang et al., 2003, 2004) correlates with the dynamics of FS (smaller versus larger structures) and, therefore, rapid cell migration. AM induces a reduction in FS size in Mv1Lu but not in HaCaT cells in a JNK-dependent mechanism (Bernabe-Garcia et al., 2017; Figure 2A). Time-lapse migration studies could help elucidate whether AM-stimulated Mv1Lu are fast migrating cells versus HaCaT. Bernabe-Garcia et al. (2017) also showed that AM induces an increase in the number of FS. Since Paxillin FS are triggered by integrin clustering, an enhanced engagement of integrin clustering could be at work (Parsons et al., 2010). On the other hand, integrin clustering activates cell signaling events intracellularly, which are coincident with those of growth factors binding to its receptor (Ivaska and Heino, 2011). Cooperation and amplification of cell signaling between these two receptor families may happen at different levels, for example, through the activation of FAK (Ivaska and Heino, 2011; Figure 2A). Indeed, there is evidence that AM induces the phosphorylation of FAK (Bernabe-Garcia et al., 2017), reinforcing the role of AM on cell migration through FS dynamics (Figures 2A,C).

### Role of AM in Proliferation

Wound fluid derived from diabetic foot and venous leg ulcers is rich in proinflammatory cytokines, such as TNF-α and IL-1β; and TGF-β (Harris et al., 1995; Jude et al., 2002). The effects of TGF-β on full-thickness wound re-epithelialization have been studied in mouse models overexpressing TGF-β at the epidermis, which causes a decrease in re-epithelialization (Yang et al., 2001; Chan et al., 2002). TGF-β induces G1 cell growth arrest of epithelial cells, and this effect is mediated by *CDK2B* (p15) and *CDKN1A* (p21) (Datto et al., 1995; Reynisdottir et al., 1995). Keratinocytes’ stimulation with TGF-β prevents cell proliferation in a mechanism that involves Smad3 (Ashcroft et al., 1999). Indeed, the downregulation of Smad3 has been suggested as a possible way of improving wound healing (Ashcroft and Roberts, 2000). Alcaraz et al. (2015) studied the relationship between TGF-β signaling and AM stimulation on HaCaT cells. In these studies, AM was able to attenuate TGF-β-induced phosphorylation of both Smad2 and Smad3, leading to the diminished expression of both *CDKN1A* (p21) and *CDK2B* (p15) and the return of cells to proliferation (Alcaraz et al., 2015; Figure 2B). AM attenuation of TGF-β pathway may be mediated by MEK activation, since the inhibition of MEK ceases this effect (Ruiz-Canada et al., 2017). Thus, AM may therefore counteract G1 cell cycle arrest induced by TGF-β on keratinocytes, releasing them from the brake imposed by TGF-β (Alcaraz et al., 2015; Figures 2B,C). Indeed, from a keratinocyte perspective, AM interfering with TGF-β signaling may be a good way to resume full keratinocyte cell proliferation in the scenario of chronic wound healing (Liarte et al., 2020b).

## Wound Re-Epithelialization by AM in Chronic Wounds: The *in vivo* Correlation

When the skin is injured, a key part of healing progression is the re-epithelialization phase. This phase starts as early as 24 h after injury, due to the fact that keratinocytes need to be activated in order to proceed with migration (Coulombe, 1997). During cell migration, keratinocytes remain part of a cohesive cell sheet, retaining some of their intercellular connections (Rousselle et al., 2019). Those in the wound border migrate, the migrating tongue, whereas the ones further behind proliferate to sustain migration (Aragona et al., 2017; Rousselle et al., 2019; Figure 1). Molecularly, migrating keratinocytes respond by producing cytoskeletal actin fibers and assembling new adhesion complexes, together with the expression of certain integrins, MMPs, ECM components, and keratins, while the keratinocytes behind become hyperproliferative.

Mechanistically, all chronic wounds show several common features, namely, excess of proinflammatory cytokines, augmentation in proteases, increased reactive oxygen species, presence of pathogens, and presence of senescent cells, together with a deficiency of stem cells, which are often dysfunctional (Rousselle et al., 2019). The final consequence is the lack of migration of keratinocytes at the leading edge during chronification (Dickinson and Gerecht, 2016).

Amniotic membrane secretes several factors that intervene in wound healing (Barrientos et al., 2008; Litwiniuk and Grzela, 2014); among them, EGF and TGF-β can be stressed. As mentioned above, both of them contribute to keratinocyte migration (Haase et al., 2003; Ramirez et al., 2014). TGF-β is known to control keratinocyte proliferation in normal skin, whereas it is necessary for keratinocyte EMT and migration during wound re-epithelialization (Ramirez et al., 2014). There is a considerable abundance of literature dedicated to the study of the effect of TGF-β during wound healing with some seemingly contradictory results when translated to chronic wounds. Among them, we point out some common facts: (i) both TGF-β ligand and receptor expression are increased in the epidermis adjacent to wound after injury and in the leading edge of the migrating epithelial tongue (Kane et al., 1991); (ii) TGF-β ligand expression is spatially and temporarily regulated, probably indicating its dual function in keratinocyte migration and proliferation control (Werner and Grose, 2003). At the chronic wound scenario, however, the temporal and spatial distribution excess of TGF-β may result in keratinocyte cell cycle arrest (Hashimoto, 2000; Liarte et al., 2020b) and, therefore, in a halt of cell supply for their migration as well. Among the factors secreted by AM, we can find EGF (Koizumi et al., 2000). In this regard, EGFR is predominantly expressed in basal keratinocytes at the normal epidermis, where it regulates keratinocyte’s proliferation, differentiation, and migration (Nanney et al., 1996). In wounded skin, EGFR expression is upregulated in keratinocytes adjacent to the injury. There, its signaling enhances keratinocyte migration through MEK1 and ERK activation (Haase et al., 2003). The fact that AM secretes TGF-β, at very low levels (Alcaraz et al., 2013), along with factor members of the EGF family, which in turn modulate TGF-β signaling in proliferating and migrating keratinocytes, may constitute the mechanism by which re-epithelialization is resumed by AM in chronic wounds (Figure 2C).

## Concluding Remarks

The AM is a powerful therapeutic agent changing the fate of stalled (chronic) wounds. As we have recapitulated, its effect consists in inducing a powerful migratory response of keratinocytes combined with proliferative events. However, until now, several questions still remain unanswered. Due to the nature of the keratinocyte cell models available, little can be known about other circumstances that concur in the chronic wound, with which AM deals in an efficient way without hesitation. Thus, it is necessary to procure cell *in vitro* models that can recapitulate the special circumstances, or at least part of them, that take place in a chronic wound with two different purposes: (i) to better understand the whole molecular mechanisms behind the therapeutic effect of AM and (ii) to use these models to improve the application of AM or other perinatal derivatives (PnD) as a proof of concept before applying them to the patient’s chronic wound.

## Author Contributions

CR-C wrote the manuscript and prepared the figures. ÁB-G read and added ideas to the manuscript. SL read and added ideas to the manuscript. MR-V read and added ideas to the manuscript. FN corrected and edited the manuscript and the figures. All authors contributed to the article and approved the submitted version.

## Conflict of Interest

The authors declare that the research was conducted in the absence of any commercial or financial relationships that could be construed as a potential conflict of interest.
